# Expanding the Disease Network of Glioblastoma Multiforme via Topological Analysis

**DOI:** 10.3390/ijms24043075

**Published:** 2023-02-04

**Authors:** Apurva Badkas, Sébastien De Landtsheer, Thomas Sauter

**Affiliations:** Department of Life Sciences and Medicine, University of Luxembourg, 2, Avenue de l’Université, L-4365 Esch-sur-Alzette, Luxembourg

**Keywords:** glioblastoma, network analysis, topology, betweenness centrality

## Abstract

Glioblastoma multiforme (GBM), a grade IV glioma, is a challenging disease for patients and clinicians, with an extremely poor prognosis. These tumours manifest a high molecular heterogeneity, with limited therapeutic options for patients. Since GBM is a rare disease, sufficient statistically strong evidence is often not available to explore the roles of lesser-known GBM proteins. We present a network-based approach using centrality measures to explore some key, topologically strategic proteins for the analysis of GBM. Since network-based analyses are sensitive to changes in network topology, we analysed nine different GBM networks, and show that small but well-curated networks consistently highlight a set of proteins, indicating their likely involvement in the disease. We propose 18 novel candidates which, based on differential expression, mutation analysis, and survival analysis, indicate that they may play a role in GBM progression. These should be investigated further for their functional roles in GBM, their clinical prognostic relevance, and their potential as therapeutic targets.

## 1. Introduction

Glioblastoma multiforme (GBM) is one of the most frequently occurring cancers of the central nervous system, though due to its low overall prevalence, (incidence: about 3/100,000 per year) [1], it is classified as a rare disease. Despite being a subject of research for decades, the prognosis remains bleak. The median survival time for GBM patients is about 15 months [2]. While treatment options include surgery, radiotherapy, and chemotherapy, expanding the therapeutic repertoire is an urgent clinical requirement, due to the limited number of drug candidates, resistance to existing drugs, and the presence of the blood–brain barrier. 

Many GBM patients harbour mutations in one or more of most commonly mutated genes (IDH1, EGFR, PTEN, TP53, PI3K, TERT, etc. [3]), and these markers are used to guide treatments. However, there is a tremendous intra-tumoural heterogeneity and phenotypic plasticity, due to genetic, epigenetic, and microenvironmental factors that influence the presentation and prognosis, and which also underlie the different resistance mechanisms [4]. These factors result in a dynamic state where diverse cell populations exist and undergo continuous changes. Understanding and identifying the underlying mechanisms contributing to the disease is one of the key steps towards identifying new drug candidates for GBM. However, obtaining patient data for brain cancers is challenging, both due to low incidence and the difficulty of obtaining quality biopsy material. The large heterogeneity and the overall low number of patients for whom molecular data are available impair our ability to detect low-frequency driver mutations with acceptable levels of statistical significance.

Computational methods such as network analysis offer complementary, bottom-up insights into how different proteins interact to contribute to systemic perturbations in a disease. Statistical methods treat genes and other -omic entities as being independent. The network structure of specific molecular interactions can form the backbone on which disease mechanisms and therapeutic strategies can be formulated, aiding purely statistical analyses in a way of adding mechanistic insights. Network topology, the layout of nodes and edges in a network, helps to highlight, contextualise, and prioritise molecular players in a given context. Topology-based measures are being used to identify novel disease associated proteins (DAPs), disease modules, or drug candidates, among others [5]. Network topology has been shown to identify proteins involved in disease modules that had not been identified during GWAS studies [6]. Network topology-based methods have been applied extensively in GBM exploration for tasks such as biomarker discovery and patient stratification [7]. For example, network-based integration of multi-omics data based on non-negative matrix factorization was applied to lower grade glioma (LGG) and GBM, to identify clusters in the data [8]. Networks derived using single cell expression in paediatric and adult patients, as well as adult glioma-derived stem cells (GSCs), identified transcription factors and signalling proteins likely involved in cell-state transitions [9]. Single cell data were also used by Park et al. to identify 52 transcriptional regulators mediating treatments in glioblastoma xenograft models, and established a pipeline to inform therapeutic strategies [10]. However, many of these methods require specific data that may not be available for all diseases, especially rare conditions. A list of disease-associated genes or proteins is usually the starting point to construct a disease-specific network for such a topology-based analysis. However, ambiguity in defining disease-associated genes or proteins translates into variability in network construction, thereby leading to low-confidence predictions. GBM, for example, has over 3000 such associations in DisGeNET [11]. Depending on the stringency of inclusion criteria, these disease-gene lists can vary, raising questions on how to define the true network. In case of such differences, it is not clear if combining information from different sources offers improved outcomes, or if there is an advantage in analysing different topologies separately. Outcomes of network-based analyses are dependent on construction methods, which affect the size and topology of the constructed network [12]. Thus, one of the challenges is to identify DAPs while incorporating this uncertainty. There are studies that have tried to assess the impact of small changes in the topology of a network (missing or additional nodes, missing or additional links, presence of disconnected components, etc.) [13,14,15]; however, few of them used biological networks. Even among real biological networks, these perturbation studies investigate cases where interactions have been almost completely mapped, such as in *C. elegans* or *S. cerevisiae* [16,17]. For such perturbation studies, the underlying assumption is that the structure of the true network is known. However, human protein–protein or gene regulatory networks are both incomplete, and also contain false positive interactions (i.e., interactions that may be detected in vitro, but may not be present in vivo). Hence, quantification of such perturbations is challenging, due to the lack of gold standards or ground truth. An interesting study on glioma networks was reported, using differences in betweenness centrality (BC) between weighted networks constructed from healthy and tumour samples [18]. However, the study considered the networks to be of the same size and topology, differing only in edge weights. The question of changes in network topology was not addressed.

Hence, the objectives of this study were two-fold—1. Identification of disease-specific central proteins that are likely to be involved in GBM, based on background-corrected BC [19], incorporating variability in the definition of DAPs. 2. To study the effect of partial networks on the consistency of predictions. To this end, we assessed nine different GBM networks, and identified some of the most consistently central nodes across them. 

We proposed 18 novel candidates to be explored in the context of GBM, based on their biological roles, differential expression, mutation status and survival analysis. We also assessed the recoverability of these candidates in partial networks, and showed that the proposed method recovers these candidates as some of the top-ranked candidates in smaller, less complete networks. This simple, generalizable method does not require extensive quantitative integration, and could provide insights into some key, topologically important molecular players in GBM.

## 2. Results

We used background-corrected BC to highlight topologically important proteins in the GBM PPI network, and explored novel proteins that may contribute to the disease. Starting with a list of DAPs, we explored their surrounding nodes in a curated PPI network. This procedure highlights degree-unbiased, topologically critical nodes in the GBM network. However, constructing the initial disease network was a non-trivial, and non-standard task. Depending on the dataset, disease ID and quality/quantity of associated evidence for disease association, one can obtain varying seed lists. In this study, we investigated the effect of different network topologies on centrality calculation, and used varying seed lists to construct and analyse partially overlapping networks. To obtain robust candidates across the different networks, we used overlapping top-ranked proteins from across these networks to obtain a consensus list. Novel candidates were studied further using literature references, expression in TCGA datasets, mutation data, and survival information, in order to examine the likelihood of their putative role in GBM.

### 2.1. Overlapping Top-Ranking Nodes on Applying Background-Corrected Centrality Analysis across Topologically Varying Glioblastoma Networks Yields Robust Putative Glioblastoma Candidates

**Figure 1:**
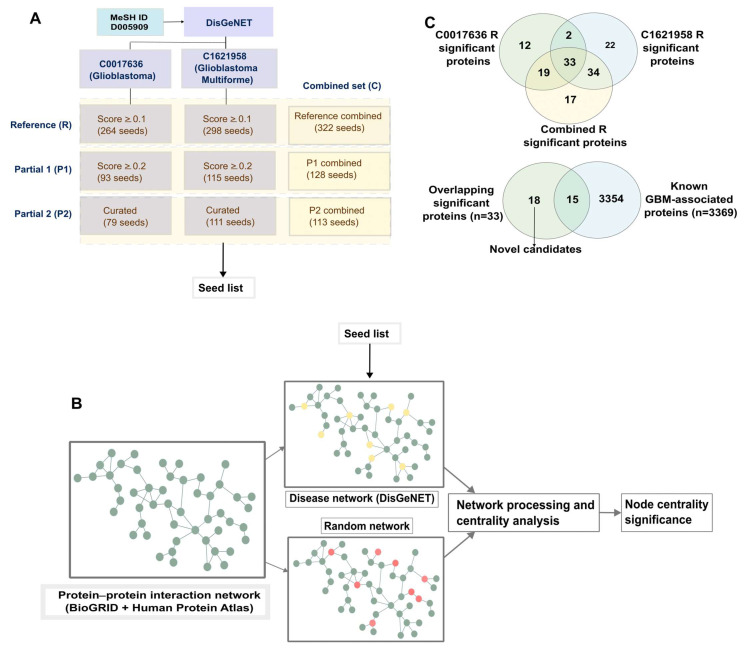
(**A**) Disease-associated proteins (DAPs) were obtained from DisGeNET. Two terms corresponding to glioblastoma and glioblastoma multiforme were used to obtain seed lists of various sizes based on different criteria. A third set of combined seed lists was also created. R: Reference (R) sets; P1: sets of DAPs with score ≥ 0.2; P2: sets of DAPs with score ≥ 0.1. (**B**) For each of the seed lists, the procedure for centrality analysis was followed—mapping seeds to PPI (yellow-DAPs, red-randomly selected nodes), creating disease-specific and degree-stratified background random networks, centrality analysis and processing, to obtain background-corrected node centralities and significance. (**C**) Overlapping significant proteins obtained from the analysis of the networks of the two categories were obtained as the most consistent candidates.

Centrality analysis for C0017636 R, C1621958 R, and the Combined R networks yielded 66, 91, and 103 statistically significant ranked proteins, respectively (Figure 1C). The overlap was of 33 proteins (Supplementary File S3 Table S6), among which 15, such as RET, EGFR, and FLT1, are already known to be associated with GBM (Supplementary File S3 Table S7). The 18 previously unlisted candidates remained significantly central across all three complete networks, and were investigated further for their role as putative novel candidates in GBM (Supplementary File S3 Tables S8 and S9). First, we established the association of these candidates with cancer. Ingenuity Pathway Analysis (Table 1 connected all 18 candidates to different processes in the context of cancer. Several functions of interest were highlighted, including the involvement of 8 out of the 18 in cell migration, 11 in necrosis and apoptosis, and others in maintenance of morphology.

Collectively, these candidates are involved in maintaining morphology, cellular development, especially neuronal development, migration, and metastasis. Based on the primary indication of cancer involvement, we examined the association of these candidates with GBM/glioma. 

### 2.2. Eighteen Novel Candidates Identified in the Study Show Links to GBM/Glioma Based on Mutations, Literature Evidence, Expression, and Survival Analysis

The biological roles of the novel candidates were explored based on mutation data, differences in gene expression, survival correlations, and pathways involved, using different datasets and databases as described in the Methods section.

**Figure 2:**
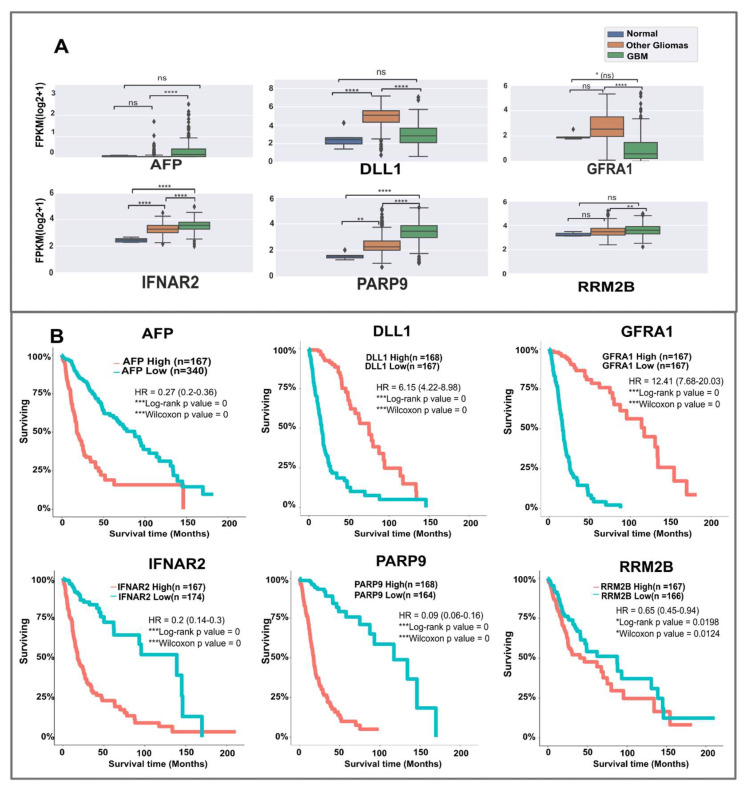
(**A**) Expressions of some candidates: level of expression on the y-axis is in terms of log of FPKM (fragments per kilobase million), and the x-axis shows the three groups: Normal (n = 5), Other gliomas (n = 445), and GBM (n = 233), from the Rahman et al. [20] study. (**B**) Survival curves for the candidates [y-axis—survival percentage, x-axis—survival time in months. Obtained and modified from glioVis [21], based on the TCGA GBMLGG dataset, GBM (n = 152), LGG (n = 515). Significance: **** *p* < 0.0001; *** *p* < 0.001; ** *p* < 0.01; * *p* < 0.05. See the Validation section of Methods for details.

We first checked for known mutations in the 18 candidates in the glioma/GBM datasets from cBioPortal. SHC2 is the most frequently mutated candidate (2%, n = 1840 patients), followed by CDC37 (1.6%), and SH3RF1 (1.1%). All the other candidates show low-frequency mutations. Next, we investigated gene expression differences. Using glioVis, two sets of data were considered for expression differences, the normal vs. GBM (TCGA_GBM) dataset, and the combined TCGA_GBMLGG dataset (Supplementary File S3 Tables S8 and S10). The candidates IFNAR2, GFRA1, PARP9, CDC37, and KALRN are significantly differentially expressed in both datasets. Expressions of candidates such as AFP, DLL1, and GRB14 were non-significant compared to normal samples; however, they were highly significant across different gliomas (Supplementary File S3 Table S10 ). To enable a comparison between normal, other glioma types, and GBM, we also plotted expression data using the processed TCGA dataset of Rahman et al. [20]. Figure 2A shows the comparison of expressions in some of the candidates between three groups—normal tissues, other gliomas, and GBM (See also Supplementary File S5 Figure S1). IFNAR2 and PARP9 show differences across all groups, while AFP and GFRA1 are significantly differentially expressed only between other gliomas and GBM. This could indicate specific contribution to the progression from lower-grade gliomas to GBM. Thus, the candidates showed mutations and expression differences in glioma/GBM patients. 

To examine if the expression differences in candidates were linked to differences in survival, we extracted survival curves for each candidate from glioVis (Figure 2B, Supplementary File S5 Figure S2). GFRA1, in particular, shows a very high hazard ratio (HR = 12.41), with higher expression correlating with better survival. Similarly, higher expressions of DLL1 (HR = 6.15) and SHC2 (HR = 5.82) are linked to higher survival probability. On the other hand, lower expressions of PARP9 (HR = 0.09), GRB14 (HR = 0.19), IFNAR2 (HR = 0.20), and AFP (HR = 0.27) correlate with better survival. Except for PSKH1, STAT4, and TLN1, all other candidates show statistically significant correlation with survival in patients. Thus, high/low expressions of these candidates are seen to correlate with glioma/GBM patient survival.

**Figure 3:**
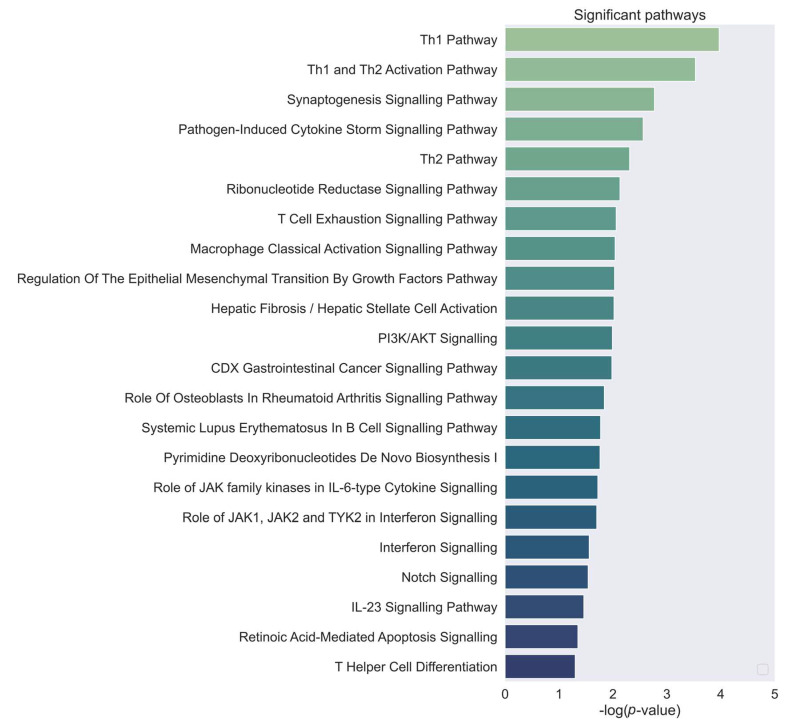
Ingenuity Pathway Analysis highlights several pathways related to the immune system, the PI3K/AKT pathway, and pathways involved in the epithelial mesenchymal transition (EMT). The name of the pathway is on the y−axis, with the significance on the x−axis.

Further hints of involvement of these candidates in invasiveness and metastasis, which are major factors contributing to the distinction between GBM and other gliomas, come from the canonical pathway analysis obtained from IPA (Figure 3). Apart from the PI3K/AKT pathway (CDC37, IL6R), Epidermal-Mesenchymal transition (IL6R, SHC2), tumour metastasis and T1/T2 activation are highlighted (DLL1, STAT4, IL6R). Murine studies indicate that the shift from the Th1 to Th2 type cell response may be a factor contributing to cancer development and progression [22]. Thus, based on mutation data, expression, survival curves, and analysis of involved pathways, the proposed candidates seem to contribute to several of the known deregulated processes in cancer, and may contribute to GBM pathology.

### 2.3. Background Correction Highlights Low-Degree Structurally Critical Proteins That Connect Several Known GBM Proteins

**Figure 4:**
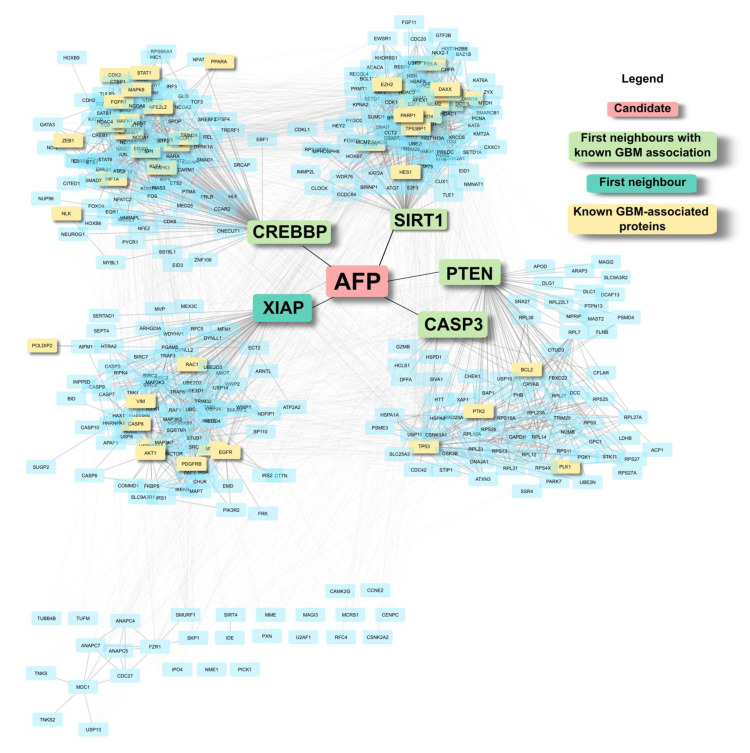
An example of AFP (in red) in the Reference-combined network. AFP is connected to five neighbours, four of which are known GBM proteins; these, in turn, are hub nodes. Clustering of the AFP network was carried out using the clusterMaker2 [23] app in Cytoscape [24].

The candidates were identified based on background-corrected BC, to highlight nodes that are central to the GBM network. To visualise these features, Figure 4 shows one of the candidates, AFP, in the Reference-combined network. AFP has five nodes as its first neighbours (degree = 5), out of which four are already known GBM-associated proteins, including PTEN. These five nodes are, in turn, hub nodes that contain large clusters with several GBM-associated proteins. The network grows from a six-node network of AFP and its first neighbours, to a network of 404 nodes and 2912 edges when expanded to first neighbours of AFP’s neighbours. As a low-degree node, it ranks much lower down the list based on raw centrality value, and may not have been highlighted without the degree correction, suggesting a critical topological position of AFP in the GBM network. 

### 2.4. Partial Networks Also Return Top-Ranked Nodes as the Top Hits, Thus Indicating the Robustness of the Method to Recover Topologically Critical Nodes

**Figure 5:**
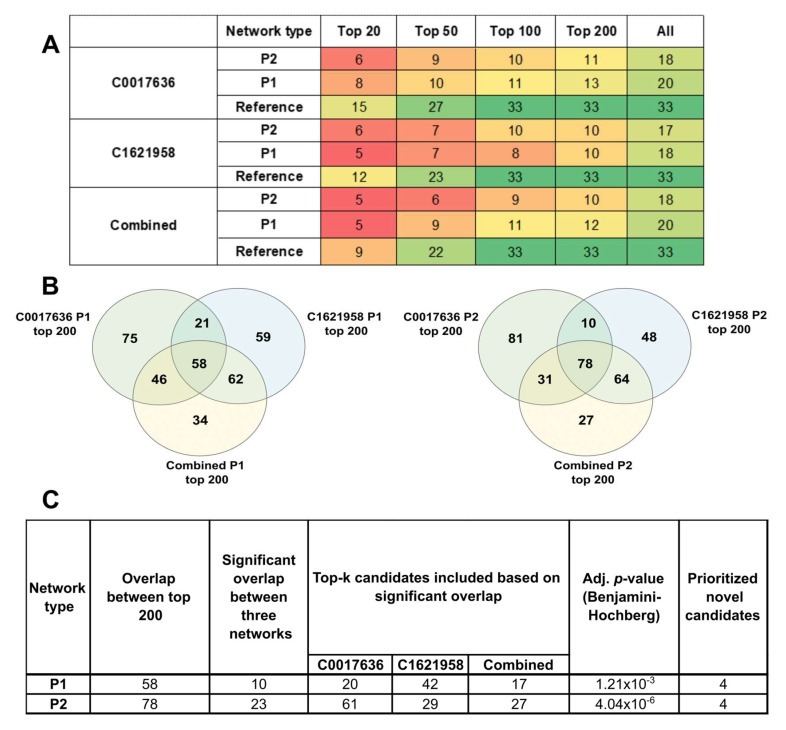
Performance of different networks considered in this study to rank significant candidates. (**A**) The table shows how many out of the 33 significant candidates are obtained in the top 20-, top 50-, and top 100-ranked nodes resulting from the network analysis pipeline. Colours range from red (lowest) to green (highest). (**B**) Overlap of top 200-ranked nodes between partial networks. (**C**) Based on the overlap obtained in (**B**), significant overlap among k-top nodes of the partial networks was obtained. This yields four consistently top-ranked candidates across all the networks.

Due to the absence of a gold-standard network, we investigated the performance of our method on partial networks, starting with varying seed lists. Indeed, BC can be sensitive to topological variations, complicating the choice between combining all available information, or performing separate analyses. To obtain a comparison between the networks, we looked at the presence of the 33 significant candidates in the top 20-, top 50-, and top 100-ranked nodes in each of the networks (Supplementary File S3 Table S6). We see from Figure 5A that the consensus networks returned fewer significant candidates as their top-ranked results than the partial networks individually. For example, the combined Reference network returned only 9 significant candidates in the top 20, as compared to C0017636 Reference (15 candidates) and C1621958 (12 candidates). Thus, in terms of capturing topologically significant nodes among its top ranks, consensus networks do not seem to perform as well as the individual networks.

To ascertain the ability of the pipeline to highlight significant nodes amongst its top ranks, we considered the varying lengths of individual networks for their enrichment. We can see that in the partial networks P1 and P2, among the significant candidates that can be found in the top 100 ranks of each network, almost 60–70% of them are present in the top 20. In the larger reference networks, 60–80% can be recovered in the top 50. Thus, the pipeline enriches the candidates among the top ranks, and seems to accommodate missing information and differences in topologies in partial networks.

To further account for the varying network sizes and the ability of partial networks to recover the novel candidates, we used the tool DynaVenn to obtain the most significant overlaps among the ranked lists, as it uses flexible thresholds for comparison among top-k members of lists. For the partial networks P1 and P2, for the top 200-ranked nodes for C0017636, C1621958, and the combined networks, 58 proteins were common between P1, and 78 among the P2 networks (Figure 5B). For each network category, DynaVenn considered varying top-k nodes, as shown in Figure 5C, and calculated the most significant overlap to be 10 and 23 nodes, respectively. Four out of the eighteen proposed candidates are top-ranking nodes across all the partial networks. These conservative results can be considered robust—these candidates are consistently top-ranked across different topologies, and after multiple-testing correction. Thus, this study highlights four proteins—SH3RF1, IFNAR2, GFRA1, and SHC2—as high-confidence GBM-associated proteins, and an expanded set of 18 probable candidates to be explored further to establish their roles in GBM.

## 3. Discussion

Using a specific PPI network analysis method and sets of known proteins associated with GBM, we confirmed 15 known GBM proteins, and proposed 18 novel candidates of interest in GBM based on mutational and expression data, survival curves, and pathway analysis. Among the confirmed proteins, RET, an oncogene, is known to play a role in the development of the central and peripheral nervous system, and EGFR is one of the most frequently mutated genes in glioblastoma. Among the novel candidates, several are enzymes (DLL1, PARP9, RRM2B, SH3RF1, USP53), three are membrane receptors (GFRA1, IFNAR2, IL6R), two are kinases (KALRN, PSKH1) and some others with miscellaneous roles, such as transcriptional regulation, and adaptor proteins (SH3RF1, SHC2)(Supplementary File S3 Table S9). We obtained a collated overview of the biological functions of the candidates from various databases, such as GeneCards [25], The National Center for Biotechnology Information (NCBI, https://www.ncbi.nlm.nih.gov/, accessed on 14 October 2022, HPA, etc. IFNAR2, IL6R, and GFRA1 are receptors for ligands linked to GBM, namely type I interferons, IL6, and RET. Several candidates are members of immune system response machinery (IFNAR2, IL6R, STAT4), while some such as PARP9 are involved in interferon antiviral response, along with functions in DNA repair. RRM2B is involved in metabolism and hypoxia response, induced due to DNA damage [Genecards]. SHC2 and GRB14 are both linked to metabolism-linked cellular growth, and belong to SHC and GRB families; other members have been annotated as glioma pathway members [KEGG pathways, [26]. KALRN, EIF1AD, DLL1, SH3RF1, and TLN1 are involved in neuronal structural integrity, plasticity, differentiation, and may contribute to the highly invasive nature of GBM as compared to other gliomas. EPB41L5 has been linked to metastasis and EMT in different cancers [NCBI]. USP53 has also been shown to play a role in mesenchymal transition [27]. In adults, AFP is a tumour marker. CDC37 is linked to stabilisation of proteins by acting as chaperone. Three of these candidates, IFNAR2, IL6R, and RRM2B, are known drug targets. These nodes could be good candidates for targeting, as they are likely to have lower side effects due to their low degree.

We had previously noted that different sizes and topologies of networks are possible based on different inclusion criteria [5]. Hence, one question arises as to whether applying a stringent criterion that yields smaller networks is more useful than having large networks with more scope for discovering new candidates but with the risk of including more false positives. This study explored what happens when starting with an incomplete or partial list of disease-associated candidates, in the realistic scenario of a relatively rare condition. We expected low-degree nodes to be more sensitive to changes in network topology, and identified nodes that remain consistently top-ranked in small, partial networks. In fact, a small but well-curated list of starting seed proteins can yield some of the key nodes obtained from expanded networks, despite not being statistically significant. 

Ultimately, the usefulness of such methods depends on the insights they can provide. We find that this study indeed highlights known proteins involved in GBM, and relevant, putative candidates that are relatively unexplored in this context. However, the candidates highlighted are reflective of the network measure chosen. As we used betweenness centrality, which is larger for nodes connecting different subgraphs and translates to proteins bridging different physiological processes, the candidates show a high level of pleiotropy, with multiple roles in metabolism, immune system function, and structural integrity maintenance. Targeting such critical nodes may address the multiple dysregulations found in the tumour environment. 

Interestingly, our study also highlights developmental proteins, such as AFP and SH3RF1, along with structural proteins. It has been reported that GBMs contain heterogeneous cell populations belonging to different subtypes (astrocytomas, oligodendrogliomas, etc.), and that these cells could be undergoing phenotypic transitions from one type to another. These transitions are reflected in continuous ranges of gene expression. These transitions are also supported by markers linked to hypoxia, stemness, and quiescence [4]. The proposed candidates reflect these functions, and the temporal variation in expression across cell populations may explain the low statistical significance for these candidates in expression and survival analysis. Lastly, some of the candidates show non-significant differences between healthy and glioblastoma samples, which could be attributed to the low number of healthy samples.

Given that this method is based on a single type of input, i.e., a PPI network, and a single type of network measure, not all of the molecular contributors of GBM will be captured. Almost 400 different network measures have been proposed [28]; however, very few of them are found to be used in practice. In order to obtain the scope of application vis-a-vis different measures, this study will need to be expanded to include different measures, topologies, and network sizes. Since this method is applicable in the absence of large molecular datasets and only requires a gene list, it might be useful to explore conditions which, like GBM, are rare and/or where limited data are available. However, these network-based methods need further refinement, more measures, and combinations need to be explored before a standardised pipeline can be established. These initial results are promising, and warrant further studies on both the method as well as on the novel candidates this study highlighted in the case of GBM. 

## 4. Methods and Materials

In this study, disease-specific networks were constructed using seed DAPs and a protein–protein interaction (PPI) network. The datasets used are described below, while the computational pipeline used was as previously described [19]. Briefly, we compared, for each protein, its BC score in the disease network with the distribution of its BC scores in 10,000 degree-stratified random networks. Statistical significance of the scores was calculated using Monte Carlo non-parametric testing.

### 4.1. Obtaining Seed Lists

Glioblastoma (GBM) is assigned the unique Medical Subject Headings [29] (MeSH) ID D005909. DisGeNET [11] yields three lists, which map to the same MeSH ID but different UMLS (Unified Medical Language System) concept IDs: C0017636 (Glioblastoma, 3177 genes), C0334588 (Giant cell glioblastoma, 95 genes), and C1621958 (Glioblastoma Multiforme, 3197 genes) (Supplementary File S1 Tables S1–S3). DisGeNET is a comprehensive resource, which includes data from many resources, such as UniProt, PsyGeNET, Orphanet, the CGI, CTD (human data), ClinGen, etc. Among the two largest sets, 2910 genes are common, while the third one is contained in both of these sets. Hence, this study was based on the two largest sets, C0017636 and C1621958, a combination of which consisted of 3369 unique genes (Supplementary File S1 Table S4). The protein products of the seed genes were termed GBM DAPs. This combined list was used as a basis for labelling proteins as known disease associations, and candidates not included were termed novel.

For the two UMLS IDs, we used 3 levels of evidence for the inclusion of seeds: (1) a manually curated list of DAPs, (2) DAPs that had an DisGeNET score ≥ 0.2, and (3) DAPs with a score ≥ 0.1. These thresholds were chosen heuristically to obtain small networks that contained the best-known glioblastoma-associated genes with reasonably sound evidence. A third set of seed lists was generated by combining the seeds across each of the selection criteria (i.e., combining curated seeds from C0017636 and C1621958, and across the other two thresholds) (Supplementary File S2 Table S5). The remaining ~2700 DAPs with low scores were not considered as seeds for this analysis. Among the nine networks, we considered the largest network of each category to be the complete reference network for that category. The other smaller networks were treated as partial networks. Figure 1A lists the initial sizes of the nine networks.

### 4.2. Mapping to PPI

Protein–protein interaction data were obtained from the multi-validated human dataset of the BioGRID [30] database (v 4.4.204). A list of 16,227 proteins that are found to be expressed in the brain (obtained from the Human Protein Atlas (HPA [31]) was used to filter the PPI network. A final PPI set of 87,424 interactions was obtained. DAPs were mapped to the processed PPI network.

### 4.3. Computational Pipeline and Resources

The centrality analysis and background correction were as described previously [19], depicted in Figure 1B. The pipeline was built in Python 3, using NetworkX for centrality computations. For each of the disease networks, the centrality score was compared against 10,000 background random networks. Networks were processed on the High-Performance Computing cluster of the University of Luxembourg [32]. The code is available on GitHub at the following address: https://github.com/sysbiolux/Background_corrected_network_analysis, accessed on 14 October 2022.

### 4.4. Overlap Significance

The significance of overlaps between the top-ranked candidates of the different partial networks was analysed using DynaVenn [33] (https://ccb-compute.cs.uni-saarland.de/dynavenn/, accessed on 14 October 2022). This online tool computes the significance of overlap between up to 3 lists. Overlaps were calculated among the top 200 proteins of the 3 networks for P1 and P2 categories. 

### 4.5. Validation

Overlapping top-ranking candidates from the networks were explored in the context of GBM, gliomas, and their roles in different cancers based on the literature. For the significant candidates, mutation data were obtained from the cBioPortal [34] database (https://www.cbioportal.org/, accessed on 7 September 2022) (Supplementary File S3 Table S8). Disease involvement information was obtained from the HPA (Supplementary File S3 Table S8). Survival analysis results were extracted from the tool glioVis [21] (http://gliovis.bioinfo.cnio.es/, accessed on 14 November 2022), using the GBM and lower-grade glioma (GBMLGG) and glioblastoma (GBM) datasets from The Cancer Genome Atlas (TCGA) [35]. The evidence table in Supplementary File S3 (Table S8) lists both outcomes, while survival curves in Figure 2B and Supplementary File S5 Figure S2 are based on the combined GBMLGG dataset. The GBM dataset compares normal samples with GBM, while the combined dataset contains GBM and other lower-grade gliomas (LGG)—oligodendroglioma, oligoastrocytoma, and astrocytoma, while normal samples are absent. Differential expression information was obtained from two sources: TCGA GBM dataset via glioVis (Supplementary File S3 Table S8), and the TCGA data processed and published by Rahman et al. [20]. The latter dataset was split into three groups: normal, GBM, and other gliomas. Samples labelled as IDH1 wild-type were considered GBM (n = 233), and all other types of gliomas were combined under ‘Other gliomas’ (n = 445). Normal samples (n = 5) were used as labelled, and these data were used to obtain the expression plots (Supplementary File S5 Figure S1). Significance was tested using the pairwise t-test, with Benjamini–Hochberg correction for multiple testing. Differential expression in the GBMLGG dataset mentioned in Supplementary File S3 Table S10 was as obtained from glioVis. Enrichment of candidates based on their known disease associations, contributions to specific biological processes, and involvement in different canonical pathways was obtained from Ingenuity Pathway Analysis (IPA, Qiagen GmbH) (Supplementary File S4 Tables S11 and S12).

## Figures and Tables

**Table 1:** Roles of the 18 identified candidates in different diseases/pathologies, as identified by Ingenuity Pathway Analysis (IPA). The table shows the name and number of the candidates from the list of 18 that participate in a given disease/pathology, the functional contribution, and the *p*-values of the association, based on the number of members of the comparison set for each disease set present in the database. Candidates are seen to play diverse roles, contributing to various processes in different functional capacities. All 18 candidates are implicated in cancer.

Diseases/Pathology	Function	*p*-Value	Candidates	Number of Proteins (/18)
Cell Death and Survival	Apoptosis	0.000115	STAT4, DLL1, TLN1, IFNAR2, GFRA1, IL6R, CDC37, SH3RF1, AFP, RRM2B, USP53	11
Cell Death and Survival, Organismal Injury and Abnormalities	Necrosis	0.000173	STAT4, DLL1, TLN1, IFNAR2, GFRA1, IL6R, CDC37, SH3RF1, AFP, RRM2B, USP53	11
Cell-To-Cell Signalling and Interaction	Activation of lymphatic system cells	0.000192	DLL1, STAT4, TLN1, IL6R, AFP	5
Tissue Morphology	Quantity of cells	0.000288	STAT4, DLL1, TLN1, IFNAR2, GFRA1, IL6R, SHC2, AFP, KALRN	9
Cell Death and Survival, Organismal Injury and Abnormalities	Cell death of tumor cell lines	0.000662	DLL1, TLN1, IFNAR2, IL6R, CDC37, SH3RF1, AFP, RRM2B	8
Cell Death and Survival, Organismal Injury and Abnormalities	Cell death of immune cells	0.000773	DLL1, STAT4, TLN1, IL6R, AFP	5
Cellular Movement	Migration of cells	0.0038	DLL1, TLN1, GFRA1, IL6R, PARP9, SH3RF1, EPB41L5, KALRN	8
Nervous System Development and Function	Morphology of nervous system	0.00591	GFRA1, IL6R, SHC2, RRM2B, KALRN	5
Cell Morphology, Nervous System Development and Function, Tissue Morphology	Morphology of neurons	0.00604	GFRA1, IL6R, SHC2, KALRN	4
Neurological Disease, Organismal Injury and Abnormalities	Progressive neurological disorder	0.0116	DLL1, IFNAR2, IL6R, RRM2B, USP53	5
Cancer, Organismal Injury and Abnormalities	Carcinoma	0.0142	STAT4, TLN1, IFNAR2, GFRA1, IL6R, SHC2, PARP9, EIF1AD, CDC37, SH3RF1, AFP, RRM2B, DLL1, EPB41L5, PSKH1, KALRN, GRB14, USP53	18

## Data Availability

Supplementary Files are included with the manuscript. Code can be found at: https://github.com/sysbiolux/Background_corrected_network_analysis, accessed on 14 October 2022.

## Supplementary Materials

The supporting information can be downloaded at: https://www.mdpi.com/article/10.3390/ijms24043075/s1.

## References

[1] Kreatsoulas D., Bolyard C., Wu B.X., Cam H., Giglio P., Li Z. (2022). Translational landscape of glioblastoma immunotherapy for physicians: Guiding clinical practice with basic scientific evidence. J. Hematol. Oncol..

[2] Mathew E.N., Berry B.C., Yang H.W., Carroll R.S., Johnson M.D. (2022). Delivering Therapeutics to Glioblastoma: Overcoming Biological Constraints. Int. J. Mol. Sci..

[3] Liu A., Hou C., Chen H., Zong X., Zong P. (2016). Genetics and Epigenetics of Glioblastoma: Applications and Overall Incidence of IDH1 Mutation. Front. Oncol..

[4] A Yabo Y., Niclou S.P., Golebiewska A. (2021). Cancer cell heterogeneity and plasticity: A paradigm shift in glioblastoma. Neuro-Oncology.

[5] Badkas A., De Landtsheer S., Sauter T. (2020). Topological network measures for drug repositioning. Briefings Bioinform..

[6] Choobdar S., Ahsen M.E., Crawford J., Tomasoni M., Fang T., Lamparter D., Lin J., Hescott B., Hu X., The DREAM Module Identification Challenge Consortium (2019). Assessment of network module identification across complex diseases. Nat. Methods.

[7] Lopes M., Martins E., Vinga S., Costa B. (2021). The Role of Network Science in Glioblastoma. Cancers.

[8] Chalise P., Ni Y., Fridley B.L. (2020). Network-based integrative clustering of multiple types of genomic data using non-negative matrix factorization. Comput. Biol. Med..

[9] Uthamacumaran A., Craig M. (2022). Algorithmic reconstruction of glioblastoma network complexity. Iscience.

[10] Park J.H., Feroze A.H., Emerson S.N., Mihalas A.B., Keene C.D., Cimino P.J., de Lomana A.L.G., Kannan K., Wu W.-J., Turkarslan S. (2022). A single-cell based precision medicine approach using glioblastoma patient-specific models. NPJ Precis. Oncol..

[11] Piñero J., Bravo À., Queralt-Rosinach N., Gutiérrez-Sacristán A., Deu-Pons J., Centeno E., García-García J., Sanz F., Furlong L.I. (2016). DisGeNET: A comprehensive platform integrating information on human disease-associated genes and variants. Nucleic Acids Res..

[12] Badkas A., De Landtsheer S., Sauter T. (2022). Construction and contextualization approaches for protein-protein interaction networks. Comput. Struct. Biotechnol. J..

[13] Niu Q., Zeng A., Fan Y., Di Z. (2015). Robustness of centrality measures against network manipulation. Phys. A Stat. Mech. Appl..

[14] Frantz T.L., Cataldo M., Carley K.M. (2009). Robustness of centrality measures under uncertainty: Examining the role of network topology. Comput. Math. Organ. Theory.

[15] Borgatti S.P., Carley K.M., Krackhardt D. (2006). On the robustness of centrality measures under conditions of imperfect data. Soc. Networks.

[16] Iyer S., Killingback T., Sundaram B., Wang Z. (2013). Attack Robustness and Centrality of Complex Networks. PLoS One.

[17] Martin C., Niemeyer P. (2019). Influence of measurement errors on networks: Estimating the robustness of centrality measures. Netw. Sci..

[18] Durón C., Pan Y., Gutmann D.H., Hardin J., Radunskaya A. (2019). Variability of Betweenness Centrality and Its Effect on Identifying Essential Genes. Bull. Math. Biol..

[19] Badkas A., Nguyen T.-P., Caberlotto L., Schneider J., De Landtsheer S., Sauter T. (2021). Degree Adjusted Large-Scale Network Analysis Reveals Novel Putative Metabolic Disease Genes. Biology.

[20] Rahman M., Jackson L.K., Johnson W., Li D.Y., Bild A.H., Piccolo S.R. (2015). Alternative preprocessing of RNA-Sequencing data in The Cancer Genome Atlas leads to improved analysis results. Bioinformatics.

[21] Bowman R.L., Wang Q., Carro A., Verhaak R.G.W., Squatrito M. (2017). GlioVis data portal for visualization and analysis of brain tumor expression datasets. Neuro. Oncol..

[22] Shurin M.R., Lu L., Kalinski P., Stewart-Akers A.M., Lotze M.T. (1999). Th1/Th2 balance in cancer, transplantation and pregnancy. Springer Semin. Immunopathol..

[23] Morris J.H., Apeltsin L., Newman A.M., Baumbach J., Wittkop T., Su G., Bader G.D., Ferrin T.E. (2011). Clustermaker: A multi-algorithm clustering plugin for Cytoscape. BMC Bioinform..

[24] Shannon P., Markiel A., Ozier O., Baliga N.S., Wang J.T., Ramage D., Amin N., Schwikowski B., Ideker T. (2003). Cytoscape: A software environment for integrated models of Biomolecular Interaction Networks. Genome Res..

[25] Rebhan M., Chalifa-Caspi V., Prilusky J., Lancet D. (1998). GeneCards: A novel functional genomics compendium with automated data mining and query reformulation support. Bioinformatics.

[26] Kanehisa M., Goto S. (2000). KEGG: Kyoto Encyclopedia of Genes and Genomes. Nucleic Acids Res..

[27] Hariri H., St-Arnaud R. (2021). Expression and Role of Ubiquitin-Specific Peptidases in Osteoblasts. Int. J. Mol. Sci..

[28] Jalili M., Salehzadeh-Yazdi A., Asgari Y., Arab S.S., Yaghmaie M., Ghavamzadeh A., Alimoghaddam K. (2015). CentiServer: A Comprehensive Resource, Web-Based Application and R Package for Centrality Analysis. PLoS ONE.

[29] Rogers F.B. (1963). Medical subject headings. Bull. Med. Libr. Assoc..

[30] Stark C., Breitkreutz B.J., Reguly T., Boucher L., Breitkreutz A., Tyers M. (2006). BioGRID: A general repository for interaction datasets. Nucleic Acids Res..

[31] Pontén F., Jirström K., Uhlen M. (2008). The Human Protein Atlas—A tool for pathology. J. Pathol..

[32] Varrette S., Bouvry P., Cartiaux H., Georgatos F. Management of an academic HPC cluster: The UL experience. Proceedings of the 2014 International Conference on High Performance Computing & Simulation (HPCS).

[33] Amand J., Fehlmann T., Backes C., Keller A. (2019). DynaVenn: Web-based computation of the most significant overlap between ordered sets. BMC Bioinform..

[34] Gao J., Aksoy B.A., Dogrusoz U., Dresdner G., Gross B.E., Sumer S.O., Sun Y., Jacobsen A., Sinha R., Larsson E. (2013). Integrative Analysis of Complex Cancer Genomics and Clinical Profiles Using the cBioPortal. Sci. Signal..

[35] The Cancer Genome Atlas Research Network (2008). Comprehensive genomic characterization defines human glioblastoma genes and core pathways. Nature.

