# Supplementary material for: Expanding the Disease Network of Glioblastoma Multiforme via Topological Analysis
Source: Int J Mol Sci. 2023 Feb 4;24(4):3075. doi: 10.3390/ijms24043075 (PMC9965660; doi:10.3390/ijms24043075)
Supplement: Supplementary file 1 [file ijms-24-03075-s001.zip › List_of_Supplementary.pdf]

Expanding the disease network of Glioblastoma Multiforme via topological analysis

Apurva Badkas [1], Sébastien De Landtsheer [1], Thomas Sauter [1, *]

[1] Department of Life Sciences and Medicine, University of Luxembourg, 2, avenue de l'Université, L-4365 Esch-sur-Alzette, Luxembourg

* Corresponding author: thomas.sauter@uni.lu

List of Supplementary files:

Supplementary File S1: Disease-associated genes for 3 Unified Medical Language System (UMLS) IDs in DisGeNET – unfiltered, Complete genelist (Tables S1, S2, S3, S4)

Supplementary File S2: Curated and filtered disease-gene associations used for network construction (Table S5)

Supplementary File S3: Results

- List of 33 candidates (Table S6)
- Known candidates (Table S7)
- Novel candidates – evidence table (Table S8)
- Novel candidates – IPA analysis (Table S9)
- Differential Gene-expression – glioVis (Table S10)

Supplementary File S4: Disease involvement and pathway analysis (Tables S11 and S12)

Supplementary File S5: Supplementary figures

Figure S1 – Gene expression

Figure S2 – Survival curves
