# Supplementary material for: Expanding the Disease Network of Glioblastoma Multiforme via Topological Analysis
Source: Int J Mol Sci. 2023 Feb 4;24(4):3075. doi: 10.3390/ijms24043075 (PMC9965660; doi:10.3390/ijms24043075)
Supplement: Supplementary file 1 [file ijms-24-03075-s001.zip › Supplementary_File_S5_Supplementary_figures.pdf]

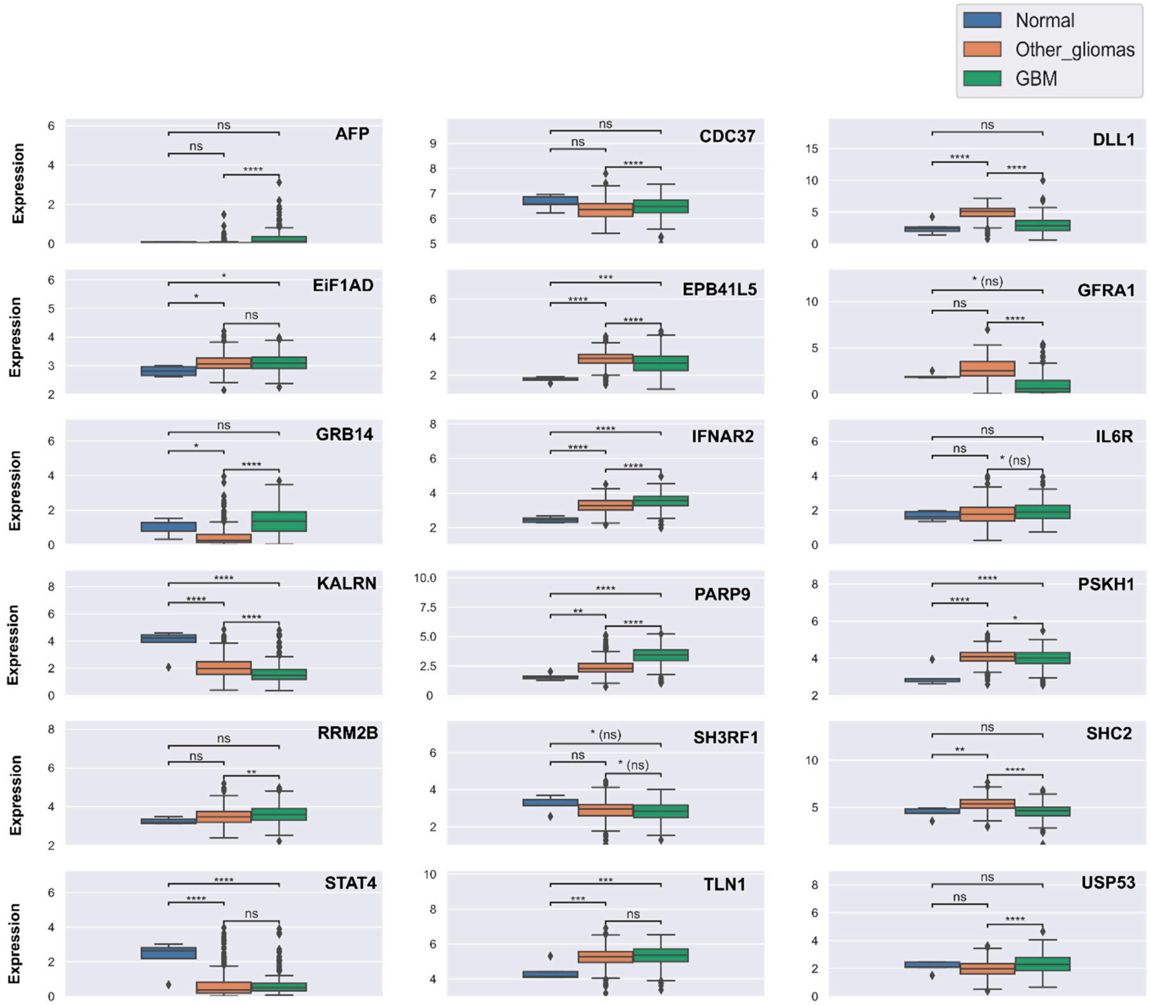

Figure S1: Expression differences between normal, other gliomas and glioblastoma (GBM) for 18 candidates. See main manuscript for details.

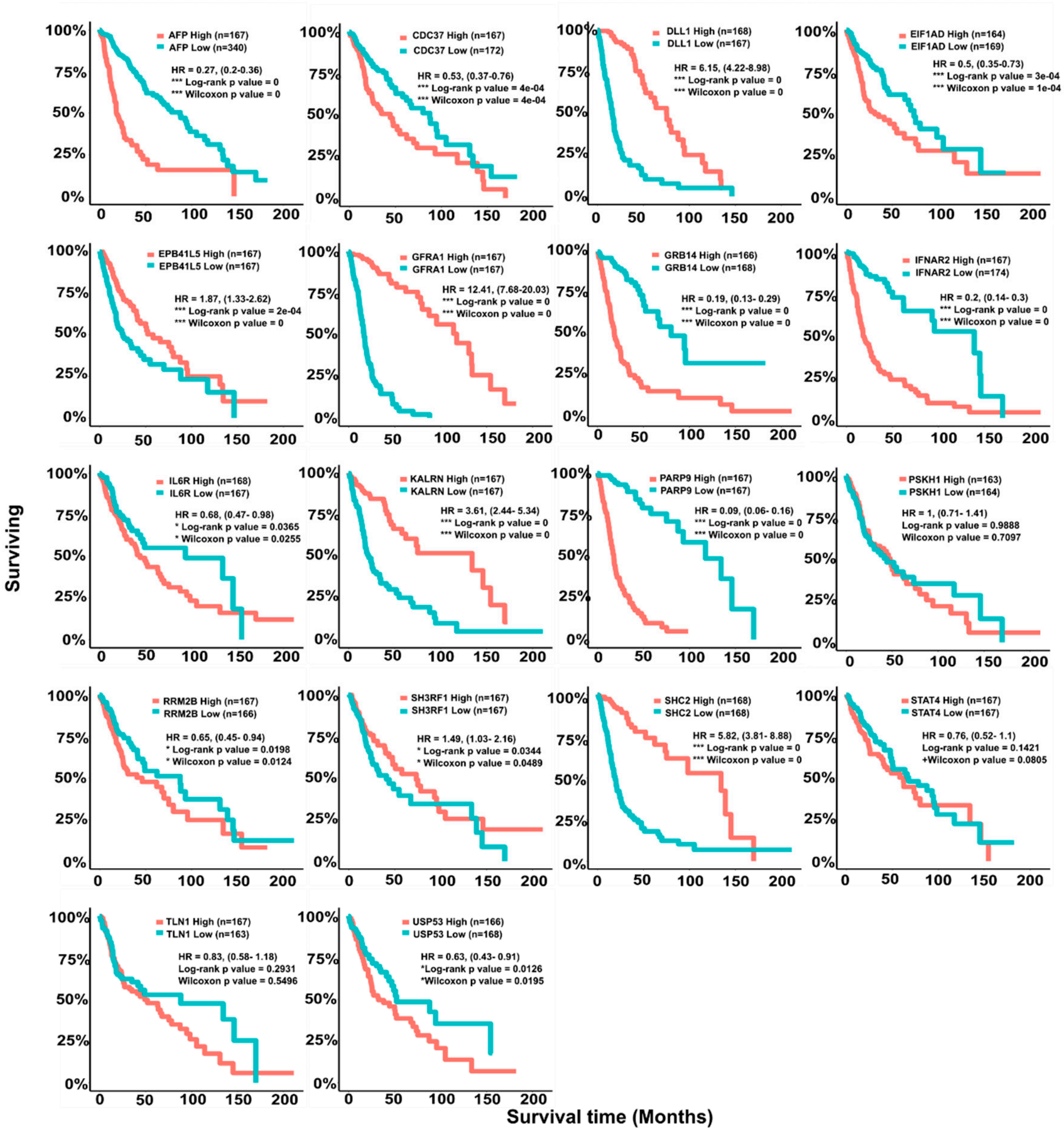

Figure S2: Survival curves for the 18 candidates. Modified from glioVis. See main manuscript for details.
